# Screening of the *SLC17A8* gene as a causative factor for autosomal dominant non-syndromic hearing loss in Koreans

**DOI:** 10.1186/s12881-016-0269-3

**Published:** 2016-01-22

**Authors:** Nari Ryu, Borum Sagong, Hong-Joon Park, Min-A Kim, Kyu-Yup Lee, Jae Young Choi, Un-Kyung Kim

**Affiliations:** Department of Biology, College of Natural Sciences, Kyungpook National University, Daegu, 41566 South Korea; Soree Ear Clinics, Seoul, 06068 South Korea; School of Life Sciences, BK21 Plus KNU Creative BioResearch Group, Kyungpook National University, Daegu, 41566 South Korea; Department of Otorhinolaryngology-Head and Neck Surgery, School of Medicine, Kyungpook National University, Daegu, 41944 South Korea; Department of Otorhinolaryngology, Yonsei University College of Medicine, Seoul, 03722 South Korea

**Keywords:** DFNA25, *SLC17A8*, VGLUT3, Autosomal dominant non-syndromic hearing loss, Mutation

## Abstract

**Background:**

One of the causes of sensorineural hearing loss (SNHL) is degeneration of the inner hair cells in the organ of Corti in the cochlea. The *SLC17A8* (solute carrier family 17, member 8) gene encodes vesicular glutamate transporter 3 (VGLUT3), and among its isoforms (VGLUT1-3), only VGLUT3 is expressed selectively in the inner hair cells (IHCs). VGLUT3 transports the neurotransmitter glutamate into the synaptic vesicles of the IHCs. Mutation of the *SLC17A8* gene is reported to be associated with DFNA25 (deafness, autosomal dominant 25), an autosomal dominant non-syndromic hearing loss (ADNSHL) in humans.

**Methods:**

In this study, we performed a genetic analysis of 87 unrelated Korean patients with ADNSHL to determine whether the *SLC17A8* gene affects hearing ability in the Korean population.

**Results:**

We found a novel heterozygous frameshift mutation, 2 non-synonymous variations, and a synonymous variation. The novel frameshift mutation, p.M206Nfs*4, in which methionine is changed to asparagine at amino acid position 206, resulted in a termination codon at amino acid position 209. This alteration is predicted to encode a truncated protein lacking transmembrane domains 5 to 12. This mutation is located in a highly conserved region in VGLUT3 across multiple amino acid alignments in different vertebrate species, but it was not detected in 100 unrelated controls who had normal hearing ability. The results from our study suggest that the p.M206Nfs*4 mutation in the *SLC17A8* gene is likely a pathogenic mutation that causes ADNSHL.

**Conclusion:**

Our findings can facilitate the prediction of the primary cause of ADNSHL in Korean patients.

## Background

Hearing loss is a relatively common defect, with an incidence of 1 to 3 per 1000 births [1, 2]. It can be classified as sensorineural hearing loss (SNHL), conductive hearing loss, or mixed hearing loss, and the major cause of hearing loss is attributed to genetic or environmental factors [3, 4]. SNHL is usually caused by malfunction or degeneration of the cochlea, including the hair cells within the organ of Corti [5, 6]. Approximately 70 % of hereditary SNHL cases are recognized as non-syndromic, which refers to hearing loss not accompanied by any clinical signs or symptoms [7–9]. Among non-syndromic hearing loss cases, approximately 75–80 % of cases are defined as autosomal recessive (DFNB), 10–20 % as autosomal dominant (DFNA), 1 % as X-linked, and less than 1 % as mitochondrial inheritance [4, 10]. Autosomal dominant non-syndromic hearing loss (ADNSHL) is genetically heterogeneous, and 33 related genes have been reported to date (http://hereditaryhearingloss.org/). Patients with ADNSHL typically have the characteristics of a late-onset age, high-frequency sensorineural hearing loss, and progressive worsening with age [11, 12]. Moreover, the causative gene of ADNSHL is considered to be a candidate gene for presbycusis [11, 13].

The *SLC17A8* (solute carrier family 17, member 8) gene associated with ADNSHL lies within the locus of DFNA25 (MIM: 605583), which is defined to be in the 20-cM region of chromosome 12q21-24 [11, 13]. The *SLC17A8* gene encodes the vesicular glutamate transporter 3 (VGLUT3) protein, which consists of 589 amino acids in 12 exons [13]. This protein is predicted to be composed of 12 transmembrane helices, which are packed in 4 groups of 3 each [14]. Each transmembrane domain is composed of 21 amino acids; the region between amino acid positions 80 and 502 is defined as the major facilitator superfamily (MFS) [15] (http://www.ncbi.nlm.nih.gov/protein/NP_647480.1). They are single-peptide secondary transporter proteins, which transport diverse hydrophilic solutes [15, 16]. There are 3 VGLUT subtypes (VGLUT1, 2, and 3), of which only VGLUT3 is expressed in the inner hair cells (IHCs) [14, 17]. The IHCs are the primary auditory sensory receptors, and glutamate is the essential excitatory neurotransmitter in the IHCs [17–20]. The main function of VGLUT3 in the IHCs is to transport this neurotransmitter into vesicles [17]. In the absence of VGLUT3 expression, the uptake and release of glutamate are disrupted at the afferent synapses of the IHCs [17–19]. Therefore, mutations in the *SLC17A8* gene would result in the malfunctioning of VGLUT3, which leads to ADNSHL [13, 17].

To date, only 1 mutation in the *SLC17A8* gene, namely, p.A211V, has been reported, which was in candidate family members of Czech and German descent in the United States [13]. These subjects had progressive, high-frequency SNHL with an autosomal dominant inheritance pattern [13]. Because no such studies on the *SLC17A8* gene in the Asian population have been reported to date, we performed gene screening in 87 unrelated Korean ADNSHL candidates and 100 unrelated Koreans with normal hearing to determine whether this gene should be considered as a contributing factor to ADNSHL in the Korean population.

## Methods

### Subjects and clinical evaluation

For this study, 87 unrelated subjects showing autosomal dominant inheritance in their pedigrees were recruited from the Department of Otorhinolaryngology-Head and Neck Surgery, Kyungpook National University Hospital in Daegu, Yonsei University Health System Hospital in Seoul, and Soree Ear Clinic in Seoul, Korea. This cohort was used in the previous genetic studies [21, 22]; these previous ones and this study were performed in the similar period. The hearing levels of the patients were examined by pure-tone audiometry (PTA). The average threshold in the PTA was measured at 500, 1000, 2000, and 4000 Hz in a sound-treated room. For PTA, the levels of hearing loss are described as follows: ~20 dB is assigned as normal hearing; 21–40 dB as mild hearing impairment; 41–70 dB as moderate hearing impairment; 71–95 dB as severe hearing impairment; and >95 dB as profound hearing impairment [23]. One hundred unrelated individuals with normal hearing were recruited from Kyungpook National University as normal controls. All the participants provided written informed consent before the study according to the protocol approved by the Ethics Committee of Kyungpook National University Hospital.

### Genetic analysis

For genetic analysis, the genomic DNA of the 87 patients and 100 controls with normal hearing was isolated from blood samples using FlexiGene DNA kits (Qiagen, Hilden, Germany). All 12 exons and intron-exon boundaries of the *SLC17A8* gene (GenBank ID: NM_139319.2) were amplified by polymerase chain reaction (PCR) using h-Taq DNA Polymerase (Solgent, Daejeon, Korea). Primers for each exon for PCR and direct sequencing were designed to be specific to their regions using Primer3web version 4.0.0 (http://primer3.ut.ee/). The amplified PCR products were then purified with exonuclease I (USB, Cleveland, OH, USA) and shrimp alkaline phosphatase (USB, Cleveland, OH, USA). Purified PCR products were then sequenced with the BigDye Terminator version 3.1 Cycle Sequencing Kit (Applied Biosystems, Foster City, CA, USA). The products were purified by ethanol precipitation and assayed with 3130*xl* Genetic Analyzer (Applied Biosystems, Foster City, CA, USA). The observed data were analyzed using Sequencing Analysis version 5.2 (Applied Biosystems, Foster City, CA, USA) and Chromas Lite version 2.1.1 (Technelysium Pty Ltd., Tewantin, QLD, Australia) software. Multiple sequence alignment was performed to compare the amino acid sequences of different vertebrate species, including *Homo sapiens, Aotus nancymaae, Bos taurus, Mus musculus, Rattus norvegicus, Xenopus tropicalis,* and *Danio rerio,* using CLC Sequence Viewer version 6.9 (CLC Bio, Aarhus, Denmark). The novelty of the newly detected variants was investigated using the dbSNP database (http://www.ncbi.nlm.nih.gov/snp/) and the 1000 Genomes Project Database (http://www.1000genomes.org/) as references. Further prediction of the pathogenicity was performed using the Mutation Taster program (http://www.mutationtaster.org).

### Restriction fragment length polymorphism analysis

To confirm whether the variations were present in the 100 normal controls, we performed a restriction fragment length polymorphism (RFLP) analysis. The restriction enzymes FokI (Takara Bio Inc., Otsu, Shiga, Japan) and PshAI (New England Biolabs Inc., Beverly, MA, USA) were chosen using SNP-RFLPing (http://bio.kuas.edu.tw/snp-rflping2/rflpUI.jsp) and Webcutter version 2.0 (http://rna.lundberg.gu.se/cutter2/). Each product was treated with each of the restriction enzymes, and they were incubated at 37 °C for 1 h to activate the enzyme. After 1 h, the products were examined by electrophoresis on 2 % agarose gels.

## Results

In this study, we screened the *SLC17A8* gene in 87 patients with ADNSHL and identified a novel frameshift mutation in 1 patient, 2 non-synonymous variations in 2 patients, and a synonymous variation in 18 patients. These patients with *SLC17A8* variations were negative for other mutations in the previous studies [21, 22].

**Fig. 1:**
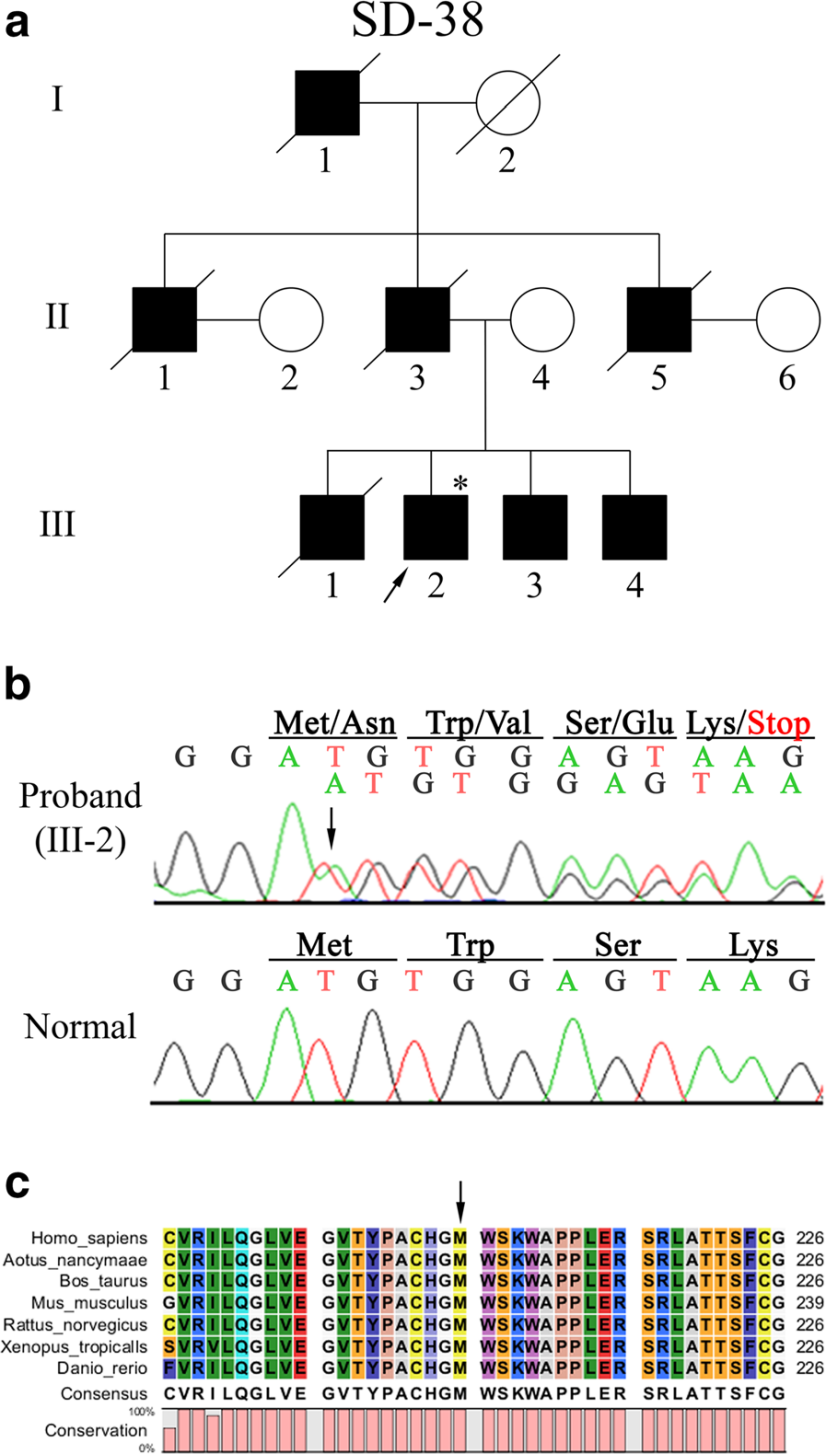
Mutation analysis of the SD-38 family with the p.M206Nfs*4 mutation. (**a**) Pedigree of Korean family SD-38. Squares and circles represent males and females, respectively. Filled symbols denote affected individuals, whereas unfilled symbols denote unaffected individuals. Diagonal lines through the symbols represent deceased members. The arrow indicates the proband of the SD-38 family. The individual evaluated in this study is marked with an asterisk. (**b**) Partial result of DNA sequencing analysis of the *SLC17A8* gene for the proband (III-2) with p.M206Nfs*4 and a normal control. The wild type nucleotide sequence at position 616 is compared with that of the proband (III-2), which shows heterozygous c.616dupA, indicated with the arrow. (**c**) Alignment of the VGLUT3 amino acid sequence in different vertebrate species: *Homo sapiens, Aotus nancymaae, Bos taurus, Mus musculus, Rattus norvegicus, Xenopus tropicalis,* and *Danio rerio*. The arrow shows the position of the p.M206Nfs*4 mutation

The SD-38 family consisted of 3 generations, with 8 affected individuals, and the pattern of inheritance was autosomal dominant (Fig. 1a). The proband (III-2) of this family was a 47-year-old man with bilateral severe hearing loss, and a novel frameshift mutation was identified at nucleotide position 616, where adenine was duplicated (c.616dupA) (Fig. 1b). The corresponding amino acid sequence produced asparagine at position 206 instead of methionine with a termination codon at amino acid position 209 (p.M206Nfs*4), which resulted in a truncated protein. The patient was heterozygous for this mutation, and no other variations were found at other exons or intron-exon boundaries. To determine whether this mutation was pathogenic only for this patient, RFLP analysis was performed since the mutation destroyed the FokI site. This variation was not found in the 100 unrelated individuals with normal hearing (data not shown). Moreover, the multiple alignments of the amino acid sequence in several vertebrate species revealed that this amino acid position 206 was highly conserved among these species (Fig. 1c).

**Fig. 2:**
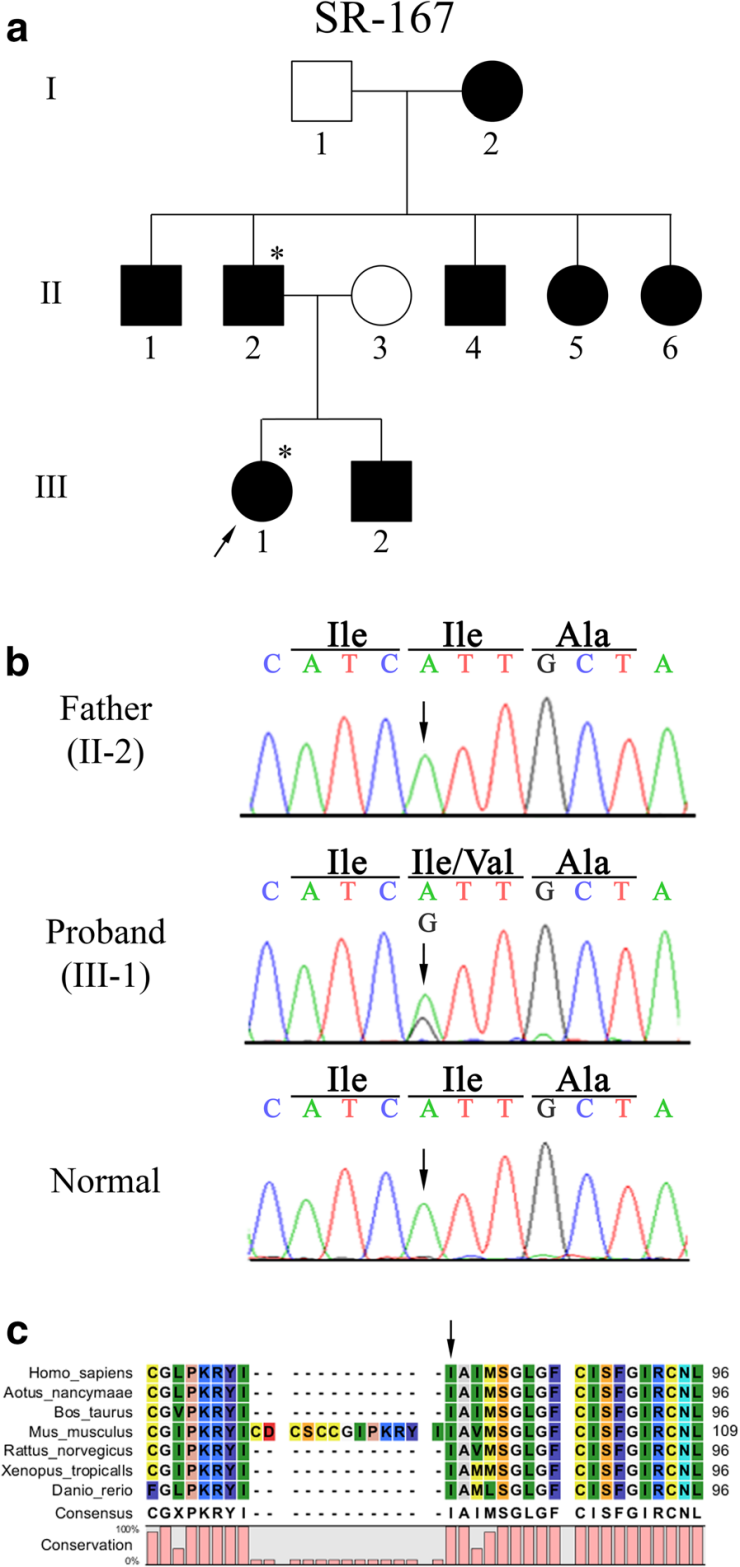
Mutation analysis of the SR-167 family with the p.I78V variation. (**a**) Pedigree of Korean family SR-167. Squares and circles denote males and females, respectively. Filled symbols represent affected individuals, and unfilled symbols represent unaffected individuals. The individuals evaluated in this study are indicated with asterisks. The proband of the SR-167 family is marked with an arrow. (**b**) Partial result of the DNA sequencing analysis of proband (III-1) , her father (II-2) and a normal control. The arrows indicate the position of c.232A > G. (**c**) Alignment of the VGLUT3 amino acid sequence in different vertebrate species: *Homo sapiens, Aotus nancymaae, Bos taurus, Mus musculus, Rattus norvegicus, Xenopus tropicalis,* and *Danio rerio*. The arrow shows the position of the non-synonymous variation p.I78V

A non-synonymous variation at nucleotide position 232 was detected in the proband (III-1) of the SR-167 family (Fig. 2a). The proband had moderate hearing loss and the average threshold in the PTA was calculated as 61.7 dB for the right ear and 70 dB for the left ear. This single-nucleotide change produced guanine at nucleotide position 232 instead of adenine (c.232A > G) resulting in an isoleucine to valine (p.I78V) substitution (Fig. 2b). This variation was reported in the 1000 Genomes Project Database (SNP ID: rs141811441) with a global minor allele frequency (MAF) of 0.1 %. However, it was predicted to be tolerated (score of 0.59) by SIFT but probably damaging (score by 0.982) by PolyPhen in the 1000 Genomes Project Database and to be disease causing by the Mutation Taster program. To verify whether this variation is pathogenic, the proband’s father (II-2) was screened. The results confirmed that this variation was not co-segregated (Fig. 2b). However, the multiple alignments of amino acid sequences showed that the amino acid position of this variation was a conserved region (Fig. 2c).

**Fig. 3:**
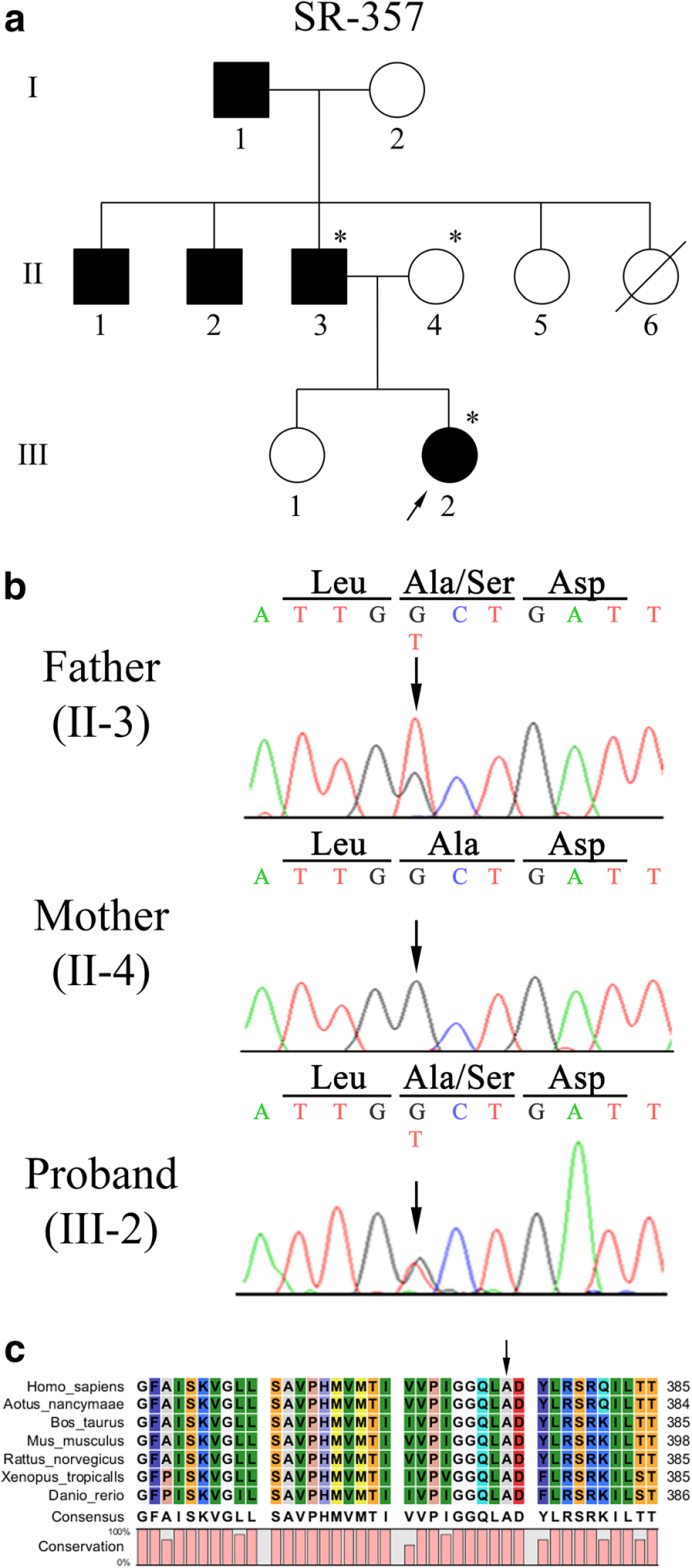
Mutation analysis of the SR-357 family with the p.A374S variation. (**a**) Pedigree of Korean family SR-357. The filled squares and the filled circle represent affected males and a female, respectively. Unfilled symbols denote unaffected individuals. The symbol with a diagonal line indicates a deceased member. The individuals evaluated in this study are denoted by asterisks. The proband of the SR-357 family is indicated with the arrow. (**b**) Partial result of the DNA sequencing analysis of proband (III-2) and her parents (II-3, II-4). The arrows indicate the position of c.1120G > T. (**c**) Alignment of the VGLUT3 amino acid sequence in different vertebrate species: *Homo sapiens, Aotus nancymaae, Bos taurus, Mus musculus, Rattus norvegicus, Xenopus tropicalis,* and *Danio rerio*. The arrow shows the position of the non-synonymous variation p.A374S

Another non-synonymous variation was identified at nucleotide position 1120 in the proband (III-2) of the SR-357 family (c.1120G > T) (Fig. 3a). This proband had moderate hearing loss calculated the average threshold in the PTA as 54.2 dB for the right ear and 45.8 dB for the left ear. The variation changed guanine to thymine, which led to an alteration in the amino acid position at 374, wherein alanine was replaced with serine (p.A374S) (Fig. 3b). This variation has been described in the 1000 Genomes Project Database (SNP ID: rs138307707) with a MAF of 0.2 %. Moreover, it has been predicted to be deleterious (score of 0.01) by SIFT, probably damaging (score of 0.999) by PolyPhen, and disease causing by the Mutation Taster program. Therefore, the genes of the proband’s parents (II-3, II-4) were screened, and the variation was revealed to be co-segregated from her father (II-3) (Fig. 3b). The position of this variation, p.A374S, was highly conserved based on the multiple alignments of amino acid sequences (Fig. 3c). However, 2 controls with normal hearing were heterozygous for this variation.

## Discussion

Degeneration or loss of function of the cochlea, including the IHCs, is an important cause of SNHL [5, 6]. VGLUT3, encoded by the *SLC17A8* gene, is expressed selectively in the IHCs [17]. The function of VGLUT3 is to transport glutamate into the synaptic vesicles, where glutamate plays an important role as an excitatory neurotransmitter [13, 15, 17].

A novel pathogenic mutation, c.616dupA (p.M206Nfs*4), in the *SLC17A8* gene was identified in a patient (III-2) of the SD-38 family. This mutation not only changed the neutral nonpolar amino acid methionine into the polar amino acid asparagine but also created a stop codon at amino acid position 209. According to molecular models, VGLUT3 is composed of 12 transmembrane helices, and position M206 is suggested to exist between transmembrane domains 4 and 5 as a topological domain and is probably located on the cytoplasmic side [15] (http://www.ncbi.nlm.nih.gov/protein/NP_647480.1). Therefore, this mutation could lead to the loss of the rest of the transmembrane domains (5 to 12), thereby resulting in a truncated protein. The truncated protein may cause malfunction or dysfunction of VGLUT3 in the IHCs, thereby leading to SNHL, but further functional studies are required to elucidate the effect of the mutation on this protein. Since neither the 1000 Genomes Project Database nor the dbSNP database had any information on this mutation and since this study is the first to describe it, we defined it as a novel mutation. Furthermore, the p.M206Nfs*4 mutation was predicted as disease causing by the Mutation Taster program. These results support the theory that the novel p.M206Nfs*4 mutation is more likely to be a pathogenic mutation causing ADNSHL.

The non-synonymous variation p.I78V in the SR-167 family was not co-segregated from the father (II-2), and the patient (III-1) may have inherited this variation from the mother (II-3), who had normal hearing. Because isoleucine and valine are neutral nonpolar amino acids, the molecular structure of the protein may not be disturbed, and the effect of the amino acid substitution may be subtle. Therefore, this variation is considered a benign polymorphism rather than a disease-causing mutation.

The single-nucleotide change in the SR-357 family was identified at amino acid position 374 (p.A374S). Although this variation was co-segregated from the father (II-3) to the proband (III-2), we found that the 2 controls with normal hearing were heterozygous for this variation. Even though a neutral nonpolar amino acid, alanine, was changed to a polar amino acid, serine, this residue may be located at the edge of transmembrane domain 8 and away from the pore according to the molecular model of VGLUT3 [15], which would have less influence on the protein structure. Therefore, it is predicted that the chance of this variation being pathogenic is relatively low. However, further functional studies will be required to confirm this hypothesis.

In addition, a synonymous variation, c.171G > A, was detected with a MAF of 1.03 % in 18 patients out of 87 subjects in this study (data not shown). This variation has been reported with a MAF of 2.8 % in the 1000 Genomes Project Database (SNP ID: rs11110359), including 1.4 % in East Asians. Our result of 1.03 % MAF was very similar to the East Asians.

## Conclusions

In summary, this study is the first genetic analysis of *SLC17A8* in patients with ADNSHL in Korea and reports a novel duplication mutation in the *SLC17A8* gene. This study will help in predicting the primary cause of ADNSHL in Korean patients. In addition, further studies with more samples are required to improve our understanding of the *SLC17A8* gene as the major causative gene for ADNSHL in Korea.

## Abbreviations

SNHL: Sensorineural hearing loss
*SLC17A8*: Solute carrier family 17, member 8
VGLUT3: Vesicular glutamate transporter 3
IHCs: Inner hair cells
ADNSHL: Autosomal dominant non-syndromic hearing loss
MFS: Major facilitator superfamily
PTA: Pure-tone audiometry
PCR: Polymerase chain reaction
RFLP: Restriction fragment length polymorphism
MAF: Minor allele frequency
